# Physical and Histological Comparison of Hydroxyapatite, Carbonate Apatite, and β-Tricalcium Phosphate Bone Substitutes

**DOI:** 10.3390/ma11101993

**Published:** 2018-10-16

**Authors:** Kunio Ishikawa, Youji Miyamoto, Akira Tsuchiya, Koichiro Hayashi, Kanji Tsuru, Go Ohe

**Affiliations:** 1 Department of Biomaterials, Faculty of Dental Sciences, Kyushu University, 3-1-1 Maidashi, Higashi-ku, Fukuoka 812-8582, Japan; tsuchiya@dent.kyushu-u.ac.jp (A.T.); khayashi@dent.kyushu-u.ac.jp (K.H.); tsuruk@college.fdcnet.ac.jp (K.T.); 2 Department of Oral Surgery, Institute of Biomedical Sciences, Tokushima University Graduate School, 3-18-15 Kuramotocho, Tokushima 770-8504, Japan; miyamoto@tokushima-u.ac.jp (Y.M.); go.ohe@tokushima-u.ac.jp (G.O.); 3 Section of Bioengineering, Department of Dental Engineering, Fukuoka Dental College, Fukuoka 814-0193, Japan

**Keywords:** carbonate apatite, hydroxyapatite, β-tricalcium phosphate, artificial bone substitute, crystallite size, dissolution rate, hybrid dog

## Abstract

Three commercially available artificial bone substitutes with different compositions, hydroxyapatite (HAp; Neobone^®^), carbonate apatite (CO_3_Ap; Cytrans^®^), and β-tricalcium phosphate (β-TCP; Cerasorb^®^), were compared with respect to their physical properties and tissue response to bone, using hybrid dogs. Both Neobone^®^ (HAp) and Cerasorb^®^ (β-TCP) were porous, whereas Cytrans^®^ (CO_3_Ap) was dense. Crystallite size and specific surface area (SSA) of Neobone^®^ (HAp), Cytrans^®^ (CO_3_Ap), and Cerasorb^®^ (β-TCP) were 75.4 ± 0.9 nm, 30.8 ± 0.8 nm, and 78.5 ± 7.5 nm, and 0.06 m^2^/g, 18.2 m^2^/g, and 1.0 m^2^/g, respectively. These values are consistent with the fact that both Neobone^®^ (HAp) and Cerasorb^®^ (β-TCP) are sintered ceramics, whereas Cytrans^®^ (CO_3_Ap) is fabricated in aqueous solution. Dissolution in pH 5.3 solution mimicking Howship’s lacunae was fastest in CO_3_Ap (Cytrans^®^), whereas dissolution in pH 7.3 physiological solution was fastest in β-TCP (Cerasorb^®^). These results indicated that CO_3_Ap is stable under physiological conditions and is resorbed at Howship’s lacunae. Histological evaluation using hybrid dog mandible bone defect model revealed that new bone was formed from existing bone to the center of the bone defect when reconstructed with CO_3_Ap (Cytrans^®^) at week 4. The amount of bone increased at week 12, and resorption of the CO_3_Ap (Cytrans^®^) was confirmed. β-TCP (Cerasorb^®^) showed limited bone formation at week 4. However, a larger amount of bone was observed at week 12. Among these three bone substitutes, CO_3_Ap (Cytrans^®^) demonstrated the highest level of new bone formation. These results indicate the possibility that bone substitutes with compositions similar to that of bone may have properties similar to those of bone.

## 1. Introduction

Bone apatite is known as carbonate apatite [CO_3_Ap: Ca_10-a_(CO_3_)_b_(PO_4_)_6-c_], which contains 6–9 wt % carbonate in an apatitic structure. However, sintered hydroxyapatite [HAp; Ca_10_(PO_4_)_6_(OH)_2_] and sintered β-tricalcium phosphate [β-TCP: Ca_3_(PO_4_)_2_] have been used in clinics as typical synthetic bone substitutes, while CO_3_Ap has not been used [1,2,3,4,5,6,7,8,9,10,11]. 

The lack of chemically pure artificial CO_3_Ap bone substitute is due to the thermal decomposition status of CO_3_Ap powder. Preparation of chemically pure CO_3_Ap powder is possible, however, CO_3_Ap powder begins to decompose at around 400 °C. Thus, CO_3_Ap blocks or granules cannot be fabricated by sintering without thermal decomposition or the release of CO_2_. This led to the invention of HAp and β-TCP, which do not contain carbonate and, thus, can be sintered. 

Sintered HAp demonstrated excellent tissue response and good osteoconductivity. Therefore, production of sintered HAp proves useful for patients. However, osteoconductivity of sintered HAp is poor when compared to that of autografts. Moreover, in contrast to autografts, which are replaced by new bone through a bone remodeling process, sintered HAp would not be replaced by new bone and remains at the bone defect. In contrast to HAp, β-TCP is resorbed at the bone defect and, thus, could be replaced by new bone. However, the dissolution rate of β-TCP is sometimes faster than the rate of bone formation, leading to unsatisfactory bone formation, or a bone defect filled with fibrous tissue.

Autograft still remains the gold standard for reconstruction of bone defects for most of the countries, even though there are serious drawbacks, including intervention at healthy sites to collect autografts. Since autograft exhibits properties superior to those of HAp and β-TCP, it is natural to develop artificial bone substitutes with compositions similar to those of bone or CO_3_Ap.

Recently, CO_3_Ap block was fabricated in aqueous solution via a dissolution–precipitation reaction, using a precursor block [12,13,14,15,16,17,18,19,20,21,22,23,24]. CO_3_Ap block upregulated differentiation of osteoblastic cells, even when compared to Hap [25]. Further, CO_3_Ap showed higher osteoconductivity than Hap [12]. CO_3_Ap block was resorbed by osteoclasts similar to bone. Thus, similar to autografts, CO_3_Ap block can be replaced by new bone [12].

Fortunately, chemically pure CO_3_Ap granules (Cytrans^®^) have become commercially available. Since CO_3_Ap block is not fabricated via sintering but through a dissolution–precipitation reaction in aqueous solution using a calcite block, the physical and histological behaviors of CO_3_Ap may be expected to be different from those of sintered HAp and sintered β-TCP. However, no comparison has been made between the commercially available bone substitutes.

In the present study, physical and histological behaviors of artificial bone substitutes were compared using three commercially available artificial bone substitutes, Neobone^®^ (HAp), Cytrans^®^ (CO_3_Ap), and Cerasorb^®^ (β-TCP).

## 2. Materials and Methods

### 2.1. Artificial Bone Substitutes

Neobone^®^ (HAp, CoorsTec, Tokyo, Japan), Cytrans^®^ (CO_3_Ap, GC, Tokyo, Japan), and Cerasorb^®^ (β-TCP, Hakuho, Tokyo, Japan), three artificial bone substitutes commercially available in Japan, were chosen for this study. 

### 2.2. Scanning Electron Microscopic Observation

Surface morphology of the three artificial bone substitutes were observed using a scanning electron microscope (SEM: S-3400N; Hitachi High Technologies Co., Tokyo, Japan) under an accelerating voltage of 15 kV, after being coated with gold–palladium. Coating was performed using a magnetron sputtering machine (MSP-1S: Vacuum Device Co., Ibaraki, Japan).

### 2.3. Compositional Analysis

Compositions of the three artificial bone substitutes were analyzed using a powder X-ray diffractometer, a Fourier transform infrared (FT-IR) spectrometer, and elemental analysis.

For X-ray diffraction (XRD) analysis, the samples were ground to fine powder, and the XRD patterns were recorded using a diffractometer (D8 Advance, Bruker AXS GmbH, Karlsruhe, Germany), generating CuKα radiation at 40 kV and 40 mA. Samples were scanned from 2θ of 10° to 40° (where θ is the Bragg angle) in a continuous mode. Crystallite size was also calculated from the XRD pattern, using Scherrer’s equation.

For FT-IR analysis, FT-IR spectra were measured using a KBr disc method, with a spectrometer (SPECTRUM 2000LX; Perkin Elmer Co. Ltd., Kanagawa, Japan).

Carbonate content was measured using elemental analysis or a CHN coder (MT-6; Yanako Analytical Instruments, Kyoto, Japan). 

### 2.4. Specific Surface Area Measurement

Specific surface areas were evaluated with a multiple Brunauer–Emmett–Teller (BET) method which evaluates nitrogen adsorption–desorption isotherms at 77 K using a BELSORP-mini II (MicrotracBEL, Osaka, Japan). 

### 2.5. Porosity Measurement

Porosity of the bone substitutes was evaluated using the formula

(1) $$\mathrm{Porosity}\;\left(\%\right)=\frac{V_{p}}{V_{p}+V_{s}}\times 100,$$

where V_p_ and V_s_ are the pore volume and the volume of the specimens, respectively. The pore volume of the bone substitutes was measured using a specific surface area analyzer. The volume of the specimens was measured by the pycnometer method, where ethanol was used as the immersion liquid.

### 2.6. Dissolution Behavior

Dissolution behavior of the three artificial bone substitutes were measured using a method defined in JIS T0330-3; Bioceramics—Part 3: Testing method of measuring dissolution rate of calcium phosphate ceramics. In short, the dissolution rate of calcium phosphate ceramics was measured by immersing in 0.08 mol/L pH 5.50 acetic acid–sodium acetate solution, and 0.05 mol/L pH 7.30 tris(hydroxymethyl)aminomethane–HCl buffer solution at 25 ± 3 °C. Sample (100 ± 1 mg) was lifted in the solution using a thread with continuous stirring, and the Ca concentration in the solution was measured using a Ca ion electrode (*n* = 5).

According to the testing method, a Ca ion-selective combination electrode (6583-10C, Horiba, Ltd., Kyoto, Japan) connected to a pH/mV-meter (Type F-72, Horiba, Ltd., Kyoto, Japan) was employed in this study.

### 2.7. Animal Experiments

Two adult male hybrid dogs (weight; 3.0–3.5 kg) were purchased from Kitayama Rabesu (Nagano, Japan). All hybrid dogs were housed in the Animal Unit of Hamri Co., Ltd. (Ibaragi, Japan) Animal protocols were reviewed and approved by the Hamri Animal Studies Committee (13-H065). The animals maintained in individual cages were provided with water and animal specific pellet-type laboratory animal food. After premedication with diethyl ether by inhalation, the animals were anesthetized with ketamine (10 mg/kg) and xylazine (3 mg/kg) via intravenous injection. Three months following the extraction of premolars and molars, a bone defect of 3.6 mm in diameter and 8 mm in depth was made in alveolar bone using an Aadva twist drill, φ3.6 mm (GC). The defect was reconstructed with Neobone^®^ (HAp), Cerasorb^®^ (β-TCP), and Cytrans^®^ (CO_3_Ap). The animals were sacrificed using intravenous pentobarbital (120 mg/kg), 4 and 12 weeks after sample implantation.

### 2.8. Histological Analysis

The specimens with surrounding tissue were carefully harvested from the mandibular bone and fixed in 10% buffered formalin for 3 days, and then dehydrated by immersion in a 70% to 100% ethanol gradient with immersion in each grade for 3 days. Finally, the bone samples were embedded in methyl methacrylate. Then, sections were prepared for pathological analysis using a modified interlocked diamond saw (Exakt, Hamburg, Germany). Then, all sections were stained with Villanueva Goldner and observed using a light microscope (DM6000B, Leica Microsystems, Heerbrugg, Switzerland). The new bone area in the defect was measured using Image J ver1.51 software (US National Institutes of Health, Bethesda, MD, USA). 

### 2.9. Statistical Analysis

For statistical analysis, one-way factorial analysis of variance (ANOVA) and Fisher’s least significant difference (LSD) post hoc test were performed using Kaleida Graph 4. Values are expressed as means ± SD. A *p* < 0.05 value was considered to indicate statistically significant differences.

## 3. Results

**Figure 1:**
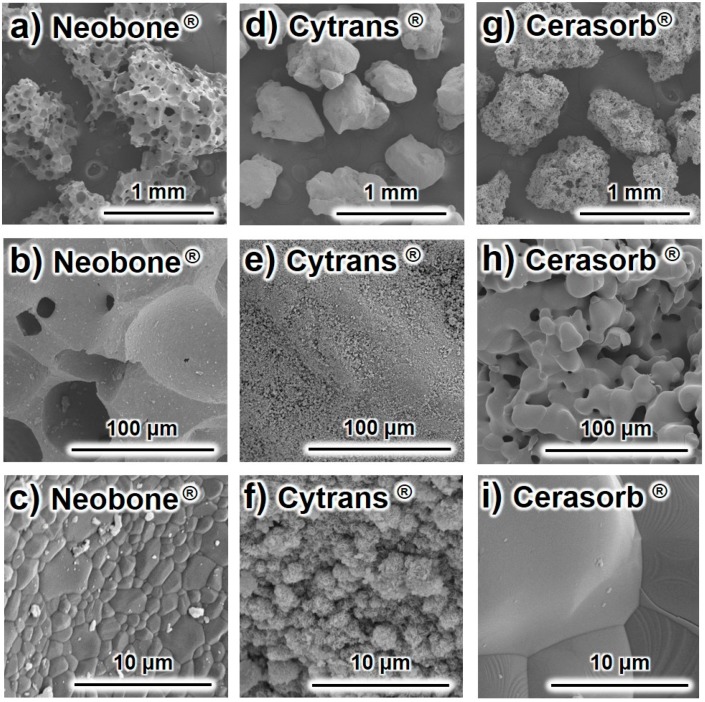
Typical scanning electron microscopy images of Neobone^®^ (HAp) (**a**–**c**), Cytrans^®^ (CO_3_Ap) (**d**–**f**), and Cerasorb^®^ (β-TCP) (**g**–**i**).

Typical SEM images of the (a–c) Neobone^®^ (HAp), (d–f) Cytrans^®^ (CO_3_Ap), and (g–i) Cerasorb^®^ (β-TCP) are summarized in Figure 1.

Both Neobone^®^ and Cerasorb^®^ displayed a porous structure, where Cerasorb^®^ had much smaller pores compared to Neobone^®^. By contrast, Cytrans^®^ was dense, and had no pores in the granules. Higher magnification showed that both Neobone^®^ and Cerasorb^®^ had smooth surfaces typical for sintering. By contrast, the surface of Cytrans^®^ was rough and consisted of small precipitated crystals.

**Figure 2:**
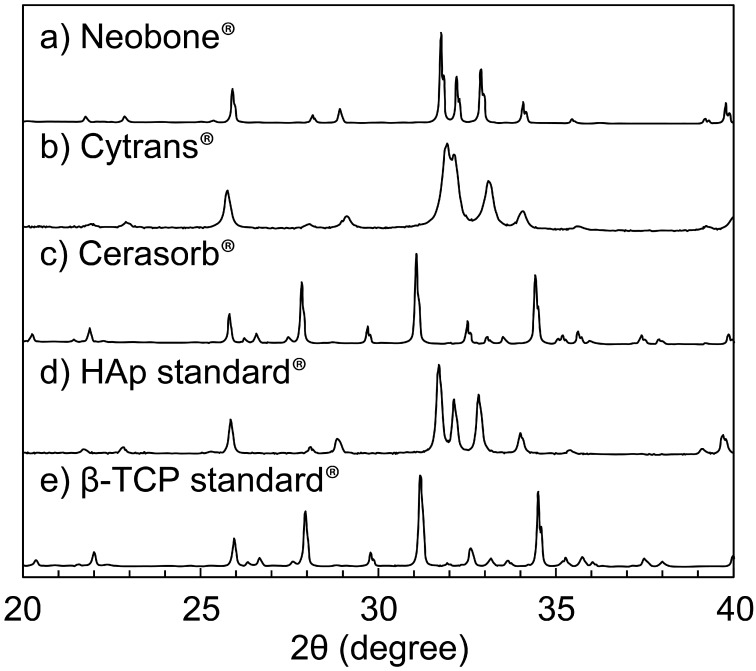
X-ray diffraction patterns of (**a**) Neobone^®^ (HAp), (**b**) Cytrans^®^ (CO_3_Ap), (**c**) Cerasorb^®^ (β-TCP), (**d**) standard Hap, and (**e**) standard β-TCP.

The XRD patterns of (a) Neobone^®^ (b) Cytrans^®^, and (c) Cerasorb^®^ are summarized in Figure 2. The XRD pattern of the standard (d) HAp and (e) β-TCP are also shown to facilitate comparison.

As shown, both Neobone^®^ and Cytrans^®^ showed typical apatitic patterns with no other peaks, whereas Cerasorb^®^ showed an XRD pattern typical of β-TCP. Peaks of the Neobone^®^ and Cerasorb^®^ were sharp, indicating high crystallinity, whereas those of Cytrans^®^ were broad, indicating low crystallinity. 

**Figure 3:**
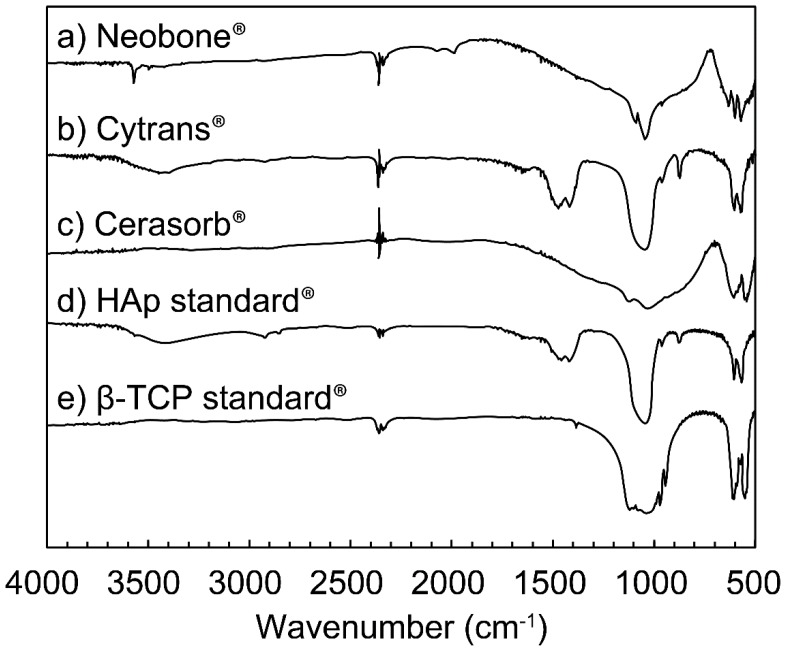
Fourier-transform infrared spectroscopy spectra of (**a**) Neobone^®^ (HAp), (**b**) Cytrans^®^ (CO_3_Ap), (**c**) Cerasorb^®^ (β-TCP), (**d**) standard Hap, and (**e**) standard β-TCP.

The FT-IR spectra of (a) Neobone^®^ (b) Cytrans^®^, and (c) Cerasorb^®^ are summarized in Figure 3. The FT-IR spectra of standard (d) HAp and (e) β-TCP are also shown to facilitate comparison.

Both Neobone^®^ and Cytrans^®^ showed typical apatitic patterns, similar to the XRD pattern. In Cytrans^®^, an absorption peak typical for CO_3_ was observed at 1450 cm^−1^, 878 cm^−1^, and 871 cm^−1^ [26,27]. No absorption peak assigned to OH was observed at 3573 cm^−1^ (ν_s_OH) and 638 cm^−1^ (ν_L_OH). By contrast, no absorption peak ascribed to CO_3_ was observed in Neobone^®^. Instead, absorption peaks ascribed to OH were observed at 3573 cm^−1^ and 638 cm^−1^. Cerasorb^®^ showed an FT-IR pattern typical of β-TCP. 

Crystallite sizes were calculated using Scherrer’s equation, and specific surface area (SSA), carbonate content, bulk density, and porosity are summarized (Table 1). Crystallite sizes of Neobone^®^ (HAp) and Cerasorb^®^ (β-TCP) were approximately 80 nm, whereas the crystallite size of Cytrans^®^ (CO_3_Ap) was approximately 30 nm, which was smaller than half of Neobone^®^ and Neobone^®^. Specific surface area was different by two orders of magnitude. SSA of Neobone^®^ (HAp) was 1.0 m^2^/g. On the other hand, SSA of Cerasorb^®^ (β-TCP) was one order of magnitude smaller than Neobone^®^ (HAp) and was 0.06 m^2^/g. By contrast, SSA of Cytrans^®^ (CO_3_Ap) was one order of magnitude larger than Neobone^®^ (HAp) and was 18.2 m^2^/g. No carbonate was found in Neobone^®^ (HAp) and Cerasorb^®^ (β-TCP), whereas Cytrans^®^ (CO_3_Ap) had 11.9 wt % of carbonate content. Cytrans^®^ (CO_3_Ap) had the largest bulk density and Neobone^®^ (HAp) had the smallest bulk density. 

As a result of bulk density and similar theoretical density, packing porosity was largest in Neobone^®^ (HAp) followed by Cerasorb^®^ (β-TCP) and Cytrans^®^ (CO_3_Ap).

**Figure 4:**
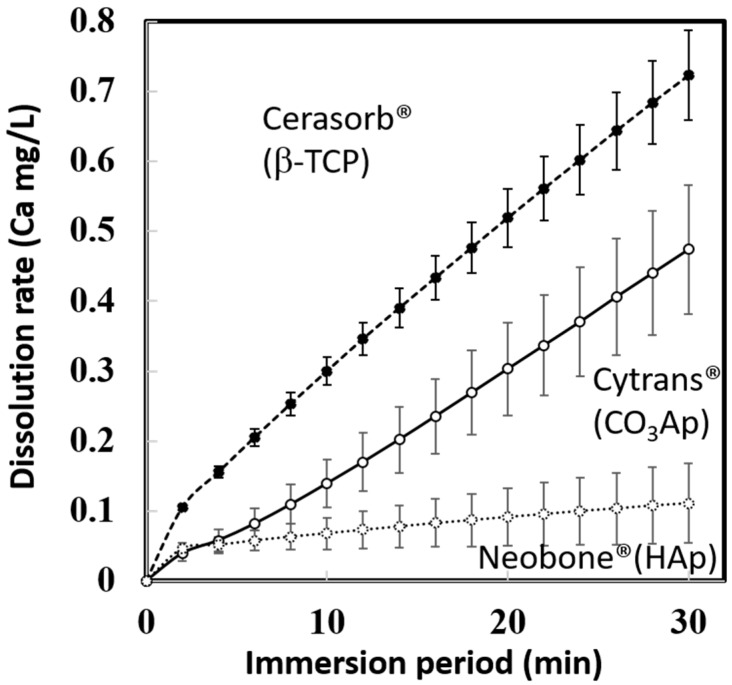
Dissolution behavior of Neobone^®^ (HAp), Cytrans^®^ (CO_3_Ap), and Cerasorb^®^ (β-TCP) in CaPO_4_-free pH 7.3 buffer solution.

The dissolution behaviors of Neobone^®^ (HAp), Cytrans^®^ (CO_3_Ap), and Cerasorb^®^ (β-TCP) in pH 7.3 CaPO_4_-free buffer solution, are summarized in Figure 4. In the neutral solution, simulating physiological condition, Cerasorb^®^ (β-TCP) showed the fastest dissolution rate followed by Cytrans^®^ (CO_3_Ap) and Neobone^®^ (HAp).

**Figure 5:**
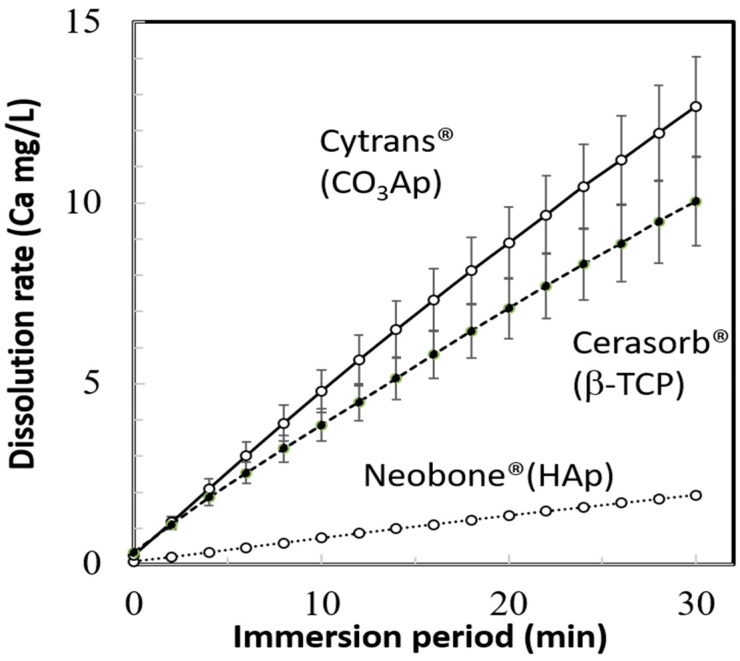
Dissolution behavior of Neobone^®^ (HAp), Cytrans^®^ (CO_3_Ap), and Cerasorb^®^ (β-TCP) in pH 5.5 buffer solution.

The dissolution behaviors of Neobone^®^ (HAp), Cytrans^®^ (CO_3_Ap), and Cerasorb^®^ (β-TCP) in pH 5.5 buffer solution are summarized in Figure 5. In the weak acidic solution, simulating the inside of the ruffle border of osteoclasts, Cytrans^®^ (CO_3_Ap) showed the fastest dissolution rate followed by Cerasorb^®^ (β-TCP) and Neobone^®^ (HAp), instead of Cerasorb^®^ (β-TCP), which showed fastest dissolution at pH 7.3.

**Figure 6:**
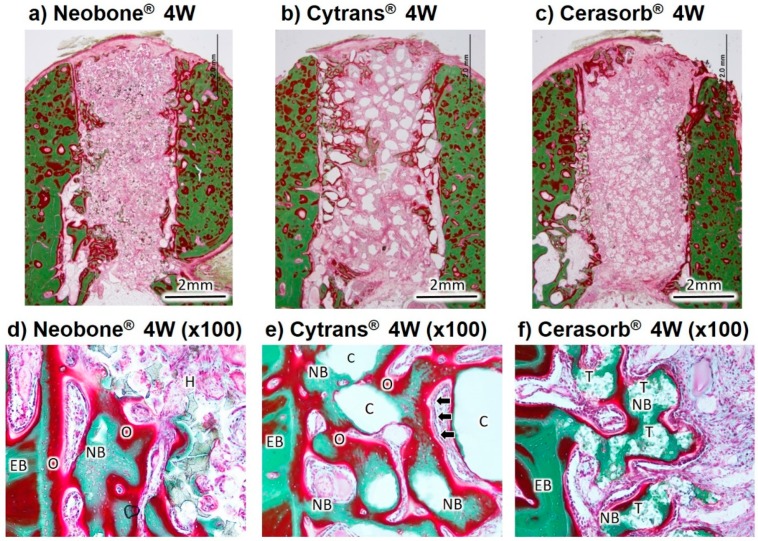
Histological findings of Neobone^®^ (HAp), Cytrans^®^ (CO_3_Ap), and Cerasorb^®^ (β-TCP) implanted into dog mandibular bone defect at 4 weeks after implantation (Villanueva Goldner staining). Green area, bone; red area, osteoid. Arrows indicate aligned cuboidal osteoblasts around the abundantly formed osteoid on the surface of the new bone. EB, existing bone; NB, new bone; O, osteoid; H, HAp (Neobone^®^); C, CO_3_Ap (Cytrans^®^); T, β-TCP (Cerasorb^®^).

**Figure 7:**
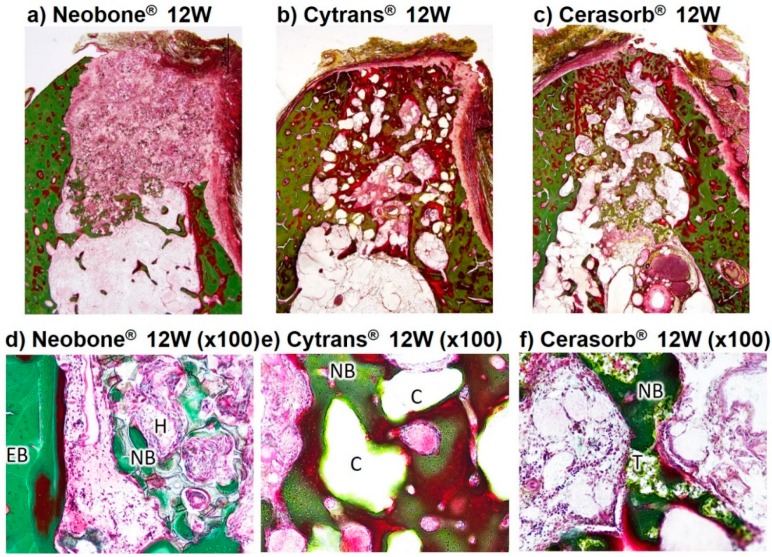
Histological findings of Neobone^®^ (HAp), Cytrans^®^ (CO_3_Ap), and Cerasorb^®^ implanted into dog mandibular bone defect at 12 weeks after implantation (Villanueva Goldner staining). Green area, bone; red area, osteoid. EB, existing bone; NB, new bone; O, osteoid; H, HAp (Neobone^®^); C, CO_3_Ap (Cytrans^®^); T, β-TCP (Cerasorb^®^).

The Villanueva Goldner-stained histological images of the three artificial bone substitutes, 4 weeks and 12 weeks after implantation, are shown in Figure 6 and Figure 7.

At 4 weeks after implantation, new bone formation was observed from existing bone to the center of the bone defect in the images of Neobone^®^ (HAp), Cytrans^®^ (CO_3_Ap), and Cerasorb^®^ (β-TCP) (Figure 6). New bone was formed on the surfaces and inside the pores of Cerasorb^®^ (β-TCP) (Figure 6f). In the central area of the defect, fibrous soft tissue had infiltrated into the interconnected porous structure of Neobone^®^ (HAp) and Cerasorb^®^ (β-TCP). The structure of Cerasorb^®^ (β-TCP) was apparently resorbed and became obscure. The new bone in Neobone^®^ (HAp) and Cerasorb^®^ (β-TCP) (Figure 6d,f) was only observed in the neighborhood of existing bone (Figure 6a). On the other hand, new bone formation in Cytrans^®^ (CO_3_Ap) had reached more central areas of the defects compared to those in Neobone^®^ (HAp) and Cerasorb^®^ (β-TCP) (Figure 6b). New bone and Cytrans^®^ (CO_3_Ap) granules were in direct contact with each other without any intermediate fibrous tissue in between (Figure 6e). Aligned cuboidal osteoblasts (arrows) were observed around the abundantly formed osteoid on the surface of the new bone, suggesting active bone formation by osteoblasts (Figure 6e). Slight chronic inflammatory cell infiltration- composed lymphocytes and plasma cells was observed in three materials (Figure 6d–f), and the strongest inflammation was recognized in Neobone^®^ (HAp) and the weakest in Cytrans^®^ (CO_3_Ap). More granulation tissue was also observed in Neobone^®^ (HAp) than Cerasorb^®^ (β-TCP) and Cytrans^®^ (CO_3_Ap).

At 12 weeks after implantation, osteoid, which was observed at 4 weeks in the image of Neobone^®^ (HAp), Cytrans^®^ (CO_3_Ap), and Cerasorb^®^ (β-TCP), was gradually replaced by mature bone. The amount of new bone in Cerasorb^®^ (β-TCP) and Cytrans^®^ (CO_3_Ap) was larger than those in Neobone^®^ (HAp) (Figure 7d–f). The granulation tissue seen at 4 weeks was replaced by fibrous tissue around Neobone^®^ (HAp), Cytrans^®^ (CO_3_Ap), and Cerasorb^®^ (β-TCP), and the inflammatory reaction had become slighter at 12 weeks compared to that at 4 weeks. Therefore, all showed good biocompatibility as evidenced by extremely slight inflammatory reaction. The size of Cytrans^®^ (CO_3_Ap) granules at 12 weeks was smaller than that at 4 weeks.

**Figure 8:**
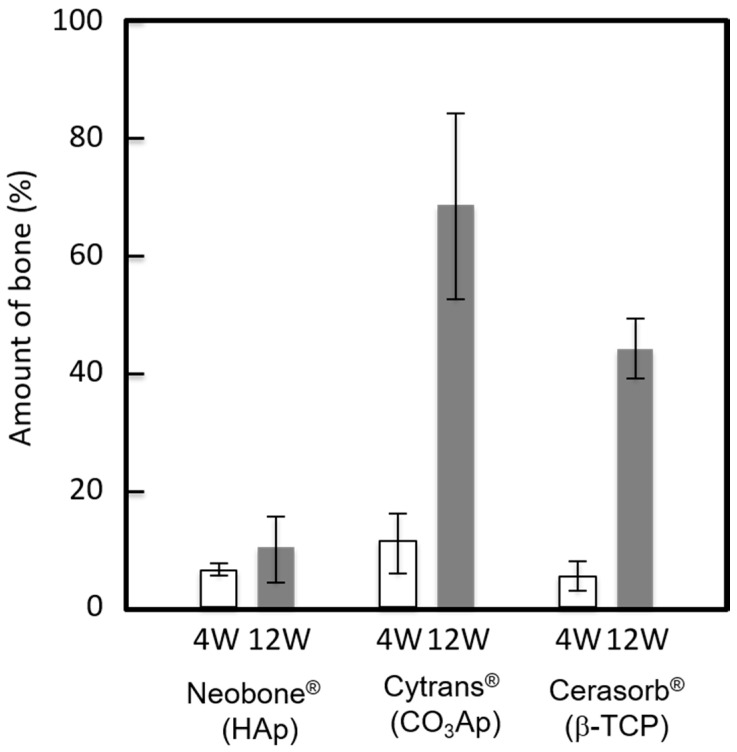
Amount of new bone formed in bone defect area at 4 weeks and 12 weeks after implantation. (*n* = 2).

The amount of bone at the bone defect 4 and 12 weeks after implantation is summarized (Figure 8). As indicated in Figure 6 and Figure 7, the largest bone amount was observed at both 4 weeks and 12 weeks in the defects reconstructed with Cytrans^®^ (CO_3_Ap). Larger amounts of bone were observed at 12 weeks compared to those at 4 weeks, regardless of the type of bone substitute.

## 4. Discussion

In this study, three commercially available bone substitutes with different composition, HAp (Neobone^®^), CO_3_Ap (Cytrans^®^), and β-TCP (Cerasorb^®^), were compared with respect to their physical character and tissue response. Sintered HAp (Neobone^®^), and sintered β-TCP (Cerasorb^®^) showed physical properties typical of ceramics fabricated by sintering. Crystallite size was large and specific surface areas were limited. When HAp (Neobone^®^) was compared with β-TCP (Cerasorb^®^), β-TCP (Cerasorb^®^) had a much smaller SSA of 0.06 m^2^/g than the 1.0 m^2^/g of HAp (Neobone^®^). Also, β-TCP (Cerasorb^®^) had smaller porosity, 76.4 ± 0.8%, compared to HAp (Neobone^®^), 85.1 ± 0.5%. 

These differences appear to be reasonable for obtaining proper dissolution behavior under physiological and osteoclastic condition. β-TCP is known to have much higher solubility than HAp, which was also confirmed in the current study using commercially available β-TCP (Cerasorb^®^) and HAp (Neobone^®^). Although the higher solubility of β-TCP is the key reason why β-TCP is replaced by new bone, β-TCP has a degree of solubility which is too high for physiological and osteoclastic pH. Therefore, Cerasorb^®^ (β-TCP) may be fabricated as it has very small SSA and small porosity, which minimizes the dissolution rate. 

CO_3_Ap (Cytrans^®^) behaves differently when compared to HAp (Neobone^®^) and β-TCP (Cerasorb^®^). It has a smaller crystallite size and a much higher SSA value. This is thought to be caused by the fabrication method, which is via a dissolution–precipitation reaction using CaCO_3_ block as a precursor in aqueous solution. Thus, crystallinity is low and crystallite size calculated from XRD pattern is smaller (30.8 ± 0.8 nm) when compared to HAp (Neobone^®^) or β-TCP (Cerasorb^®^) with crystallite sizes of 75.4 ± 0.9 nm and 78.5 ± 7.5 nm, respectively.

Dissolution of β-TCP (Cerasorb^®^) in pH 7.3 solutions was the fastest, even though it had the smallest SSA (0.06 m^2^/g), followed by CO_3_Ap (Cytrans^®^) that had the largest SSA (18.2 m^2^/g), and HAp (Neobone^®^) that had a medium SSA (1.0 m^2^/g). It should be noted that the dissolution test was done using a buffer solution containing no Ca-PO_4_, according to standards. Body fluid was supersaturated with respect to apatite. Therefore, the least dissolution of HAp (Neobone^®^) and CO_3_Ap (Cytrans^®^) should be lower than that seen in these results.

Interestingly, dissolution in the pH 5.5 solution was the fastest in CO_3_Ap (Cytrans^®^), followed by β-TCP (Cerasorb^®^) and HAp (Neobone^®^). This may due to the release of CO_2_ during dissolution in pH 5.5 solution in the case of CO_3_Ap (Cytrans^®^). Although behavior of bone substitute at the bone defect may be more complex, dissolution behavior in pH 5.5 is thought to be related to the replacement by new bone. Both CO_3_Ap (Cytrans^®^) and β-TCP (Cerasorb^®^) are known to be replaced by new bone, whereas HAp (Neobone^®^) is stable at the bone defect, and would not be replaced with bone.

Histological results, summarized in Figure 6, Figure 7 and Figure 8, revealed that bone formation and resorption of bone substitute is different according to the type of bone substitute. In HAp (Neobone^®^), new bone formation was limited in the neighborhood of existing bone 4 weeks after implantation, and the amount of new bone was very small. At 12 weeks, the amount of bone was almost the same as that at 4 weeks. 

In contrast to HAp (Neobone^®^), much larger bone was formed at both 4 weeks and 12 weeks in the case of CO_3_Ap (Cytrans^®^). Most of the CO_3_Ap granules were partially replaced by new bone at 12 weeks. Cell-to-cell signaling and material-to-cell signaling may be the cause of larger new bone formation in the case of CO_3_Ap (Cytrans^®^). In other words, osteoclasts activated by resorption may release clastokines, including TRAcP, sphingosine-1-phosphate (S1P), BMP6, wingless-type 10b (Wnt10b), hepatocyte growth factor (HGF), collagen triple helix repeat-containing protein 1 (CTHRC1), where these factors activate preosteoblasts and osteoblasts [28,29]. For material-to-cell signaling, Nagai et al. reported that CO_3_Ap upregulates osteoblastic differentiation of the human bone marrow compared to HAp [12].

β-TCP (Cerasorb^®^) showed interesting bone formation. At 4 weeks, almost no bone formation was observed. At this stage, materials remained at the bone defect. However, at 12 weeks after implantation, a larger amount of bone was formed, and most of the materials were resorbed at this stage. Although histological studies were performed only at 4 and 12 weeks, dissolution of the materials may be a key factor for β-TCP (Cerasorb^®^).

## 5. Conclusions

The CO_3_Ap (Cytrans^®^) bone substitute, which is fabricated through dissolution–precipitation reaction using a precursor block in aqueous solution, showed a much higher specific surface area and a low crystallite size when compared to sintered HAp (Neobone^®^) and sintered β-TCP (Cerasorb^®^). In acidic pH 5.3 solution mimicking Howship’s lacunae, CO_3_Ap (Cytrans^®^) dissolved most rapidly, but showed limited dissolution in physiological pH 7.3 solution, indicating stability under physiological conditions. Further, CO_3_Ap (Cytrans^®^) elicited the largest amount of new bone compared to HAp (Neobone^®^) and β-TCP (Cerasorb^®^). Composition similar to that of bone may be an advantageous factor for artificial bone substitutes.

## Figures and Tables

**Table 1:** Physical properties of Neobone^®^ (HAp), Cytrans^®^ (CO_3_Ap), and Cerasorb^®^ (β-TCP).

Bone Substitute	Crystallite Size (nm)	SSA (m^3^/g)	CO_3_ Content (%)	Bulk Density (g/cm^3^)	Porosity (%)
Neobone^®^ (HAp)	75.4 ± 0.9	1.0	-	0.47 ± 0.02	85.1 ± 0.5
Cytrans^®^ (CO_3_Ap)	30.8 ± 0.8	18.2	11.9	0.99 ± 0.03	68.7 ± 0.9
Cerasorb^®^ (β-TCP)	78.5 ± 7.5	0.06	-	0.72 ± 0.03	76.4 ± 0.8
